# Modulation of *Abortiporus biennis* Response to Oxidative Stress by Light as a New Eco-Friendly Approach with a Biotechnological Perspective

**DOI:** 10.3390/ijms26125482

**Published:** 2025-06-07

**Authors:** Anna Pawlik, Adrianna Rudawska, Anita Swatek, Grzegorz Janusz, Magdalena Frąc, Marcin Grąz, Przemysław Matuła, Magdalena Jaszek

**Affiliations:** 1 Department of Biochemistry and Biotechnology, Institute of Biological Sciences, Maria Curie-Skłodowska University, Akademicka 19 St., 20-033 Lublin, Polandgrzegorz.janusz2@mail.umcs.pl (G.J.); marcin.graz@mail.umcs.pl (M.G.); 2 Institute of Biological Sciences, Maria Curie-Skłodowska University, Akademicka 19 St., 20-033 Lublin, Poland; 3 Institute of Agrophysics, Polish Academy of Sciences, Doświadczalna 4, 20-290 Lublin, Poland; m.frac@ipan.lublin.pl; 4 Institute of Computer Science and Mathematics, Maria Curie-Skłodowska University, M. Curie-Skłodowska 1 Sq., 20-031 Lublin, Poland; przemyslaw.matula@mail.umcs.pl

**Keywords:** *Abortiporus biennis*, white rot fungi, menadione, light, oxidative stress, stress response

## Abstract

To comprehensively explore the impact of oxidative stress, induced by menadione and light at various wavelengths, on the metabolism and selected biochemical markers of the white rot fungus *Abortiporus biennis*, a phenotypic approach based on FF Panels and biochemical analysis was applied. It was possible to determine the metabolic profile of this basidiomycete, which varied greatly during fungal growth. A noticeable effect of green and red light and menadione on the overall metabolic activity and the theoretical metabolic efficiency was observed. The fungus exhibited preferences for the utilisation of polymers. The analysis of biochemical parameters revealed the highest levels of the superoxide anion radical in cultures grown in darkness and red light. The concentration of phenolic compounds in the presence of menadione slightly increased, reaching its highest level on day 10 after stress stimulation. The most substantial antioxidative effect was observed on the fifth day in cultures incubated in green light. The addition of menadione significantly stimulated laccase activity but had a negative effect on superoxide dismutase and catalase activities. In general, higher enzymatic activities were observed in white light conditions; additionally, in the case of dismutase activity, higher activities were determined in the blue and dark light variants. The findings presented in this study indicate that the biochemical changes are a resultant phenomenon of the action of the two stressors, and the response of this fungus to light- and menadione-induced oxidative stress is complex and multidirectional. These data may provide a basis for efficient and simple improvements of the industrial and medicinal potential of *A. biennis*.

## 1. Introduction

Fungi, like other organisms in the aerobic environment, are exposed to a wide range of extracellular stimuli, the effects of which may vary. Consequently, these organisms experience physical and chemical stresses, often collectively referred to as abiotic stress. The fungal response to these agents is usually a specific, intricate, and multi-level process involving responses at the morphological, physiological, biochemical, and molecular levels [1,2]. Irrespective of its nature and origin, the stress factor induces cellular oxidative stress generated as a by-product of aerobic respiration; i.e., it triggers the formation of reactive oxygen species (ROS), which include hydrogen peroxide, superoxide, and hydroxyl radicals, or lowers the antioxidative activity in the cell. These highly reactive compounds affect all cellular macromolecules, including proteins, nucleic acids, lipids, and carbohydrates [3]. It should be noted that ROS are side-products of numerous common metabolic pathways located in fungal cells. In physiological conditions, these forms are inactivated by the interaction of enzymatic and non-enzymatic antioxidants as protective mechanisms. The involvement of reactive oxygen species in intracellular signalling and communication between cells is well-documented, as is their capacity to damage cellular elements and disrupt normal cellular homeostasis [1,3]. Finally, oxidative stress substantially affects fungal cells in diverse bioproduction systems, impacting the synthesis of biotechnologically essential metabolites, such as laccase or low-molecular-weight compounds. A profound understanding of the stress responses exhibited by yeasts and filamentous fungi is imperative for the subsequent refinement of industrial processes [2,4].

One of the most significant environmental factors affecting fungal metabolism is visible light, which controls physiological adaptation, developmental decisions, morphogenesis, circadian rhythms, and stress and metabolic responses [5,6,7,8]. It is imperative to acknowledge that the light response exhibited by fungi is attributable to the integration of multiple light inputs by discrete photosensory proteins, which include flavin-based blue light, retinal-containing green light (e.g., rhodopsin), and tetrapyrolle-based red light sensors [7]. All these photoreceptors are responsible for the active reception and transduction of specific light signals across the entire spectrum of sunlight, resulting in changes at the gene expression level [9,10].

As one of the oldest organisms on Earth, fungi have evolved various sophisticated mechanisms that allow them to adapt to changing environmental conditions and cope with environmental stress [1,6,11]. Compared to unicellular fungi, filamentous fungi have additional mechanisms to deal with the processes involved in metabolising reactive oxygen species. These include a greater number of antioxidants, oxidoreductive enzymes such as catalase and superoxide dismutase, and the ability to produce secondary metabolites with antioxidant activity [12,13]. It has been shown that enzymes involved in specific pathways, such as those related to lignocellulose metabolism, including laccase, may also be involved in the multiple stress response [14].

Fungi are a diverse group, and those capable of decomposing plant-based polymers, such as lignocellulose, are of particular significance. The main organisms responsible for lignocellulose degradation are an ecological group of wood-degrading fungi, particularly those that cause white rot [15]. These organisms possess a high degree of adaptability, including the capacity to withstand toxic environmental conditions, and demonstrate effective responses to environmental stress factors, i.e., high temperatures, adverse pH, prooxidants, or heavy metals [16,17,18]. As shown in the study of [19], these properties are associated with the production of a range of enzymes, reactive oxygen species, mediators, and intermediate metabolites. The emerging potential of using fungal preparations in medicine, industry, or agrobiotechnology requires the search for new possibilities to profoundly understand their metabolism in order to manipulate their pathways to obtain high amounts of desired metabolites [4,20].

Therefore, the objective of this study is to comprehensively explore the impact of chemically caused oxidative stress induced by menadione (synthetic vitamin K) and physical stress elicited by light at various wavelengths on the metabolism and selected biochemical markers of the white rot fungus *A. biennis*. The combination of these two stress factors with environmentally friendly and non-toxic properties provides an opportunity to determine their possible synergistic effect towards modifying the biotechnological potential of the fungus.

## 2. Results

**Figure 1:**
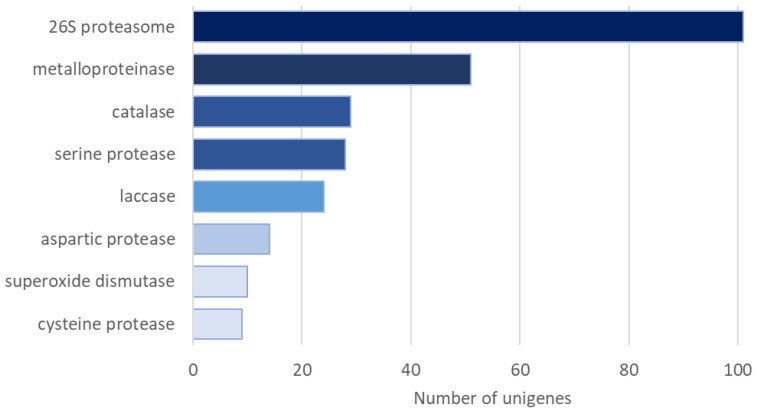
Transcripts of genes putatively involved in the response to stress factors identified in *A. biennis*. Colour bars represent the number of unigenes identified in the *A. biennis* transcriptome in the non-induced conditions. All unigenes were grouped into eight categories: 26S proteasome, metalloproteinase, serine protease, aspartic protease, cysteine protease, laccase, catalase, and superoxide dismutase.

The results of the mRNA-Seq approach performed previously for *A. biennis* cultured in non-induced conditions [21] allowed us to reanalyse the obtained data and detect the transcripts of genes putatively involved in the response to stress factors, i.e., light (physical) and menadione (chemical, oxidative stress). Based on the in silico analysis of the *A. biennis* transcriptome, several gene-encoding putative fungal photoreceptors were found (Table S1). Two of them were annotated as white-collar proteins similar to the corresponding proteins of *Neurospora crassa*. Seven genes encoded proteins similar to the cryptochrome or photolyase of *N. crassa* or bacterial strains, while the putative protein products of thirteen other genes were similar to the phytochrome of different taxa and nine to opsin. Furthermore, the analysis revealed the presence of several transcripts encoding the 26S proteasome complex (101), metalloproteinases (51), catalases (29), serine proteases (28), laccases (24), aspartic proteases (14), superoxide dismutases (10), and cysteine proteases (9) (Figure 1).

**Figure 2:**
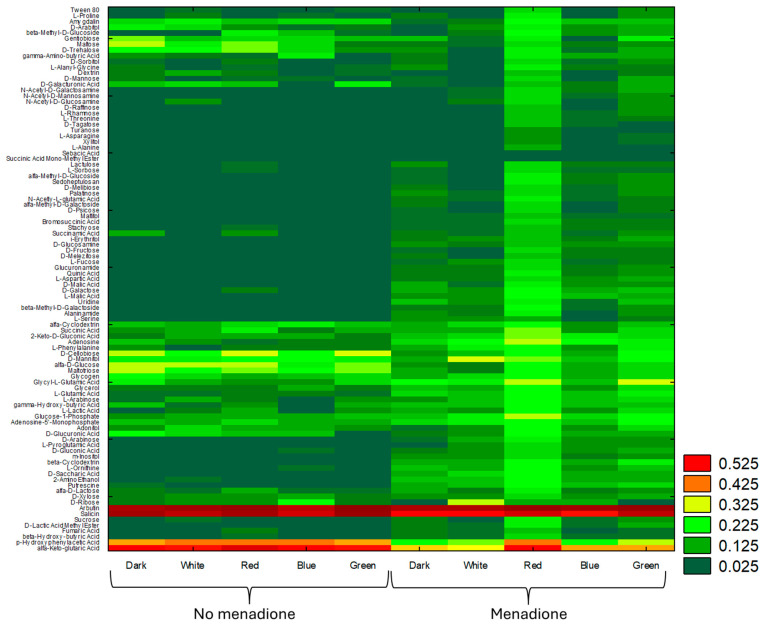
Heat map of the substrate utilisation profile of *A. biennis* cultivated in different light conditions and in the presence or absence of menadione during 192 h of incubation; the colour scale indicates mitochondrial activity measured at 490 nm for each compound.

**Figure 3:**
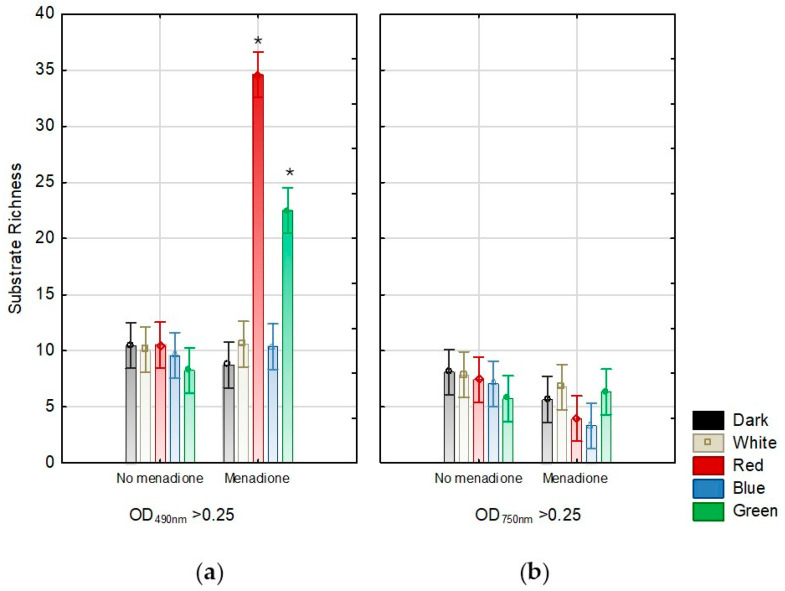
Substrate richness of *A. biennis* when grown in different light conditions and in the presence or absence of menadione measured at 490 nm (**a**) and 750 nm (**b**); vertical bars indicate 0.95 confidence intervals; bars represent means with error bars denoting standard deviation (SD) from three measurements (n = 3); and the asterisks above the result bars indicate statistically significant differences between groups for each treatment, according to Tukey’s HSD test.

**Figure 4:**
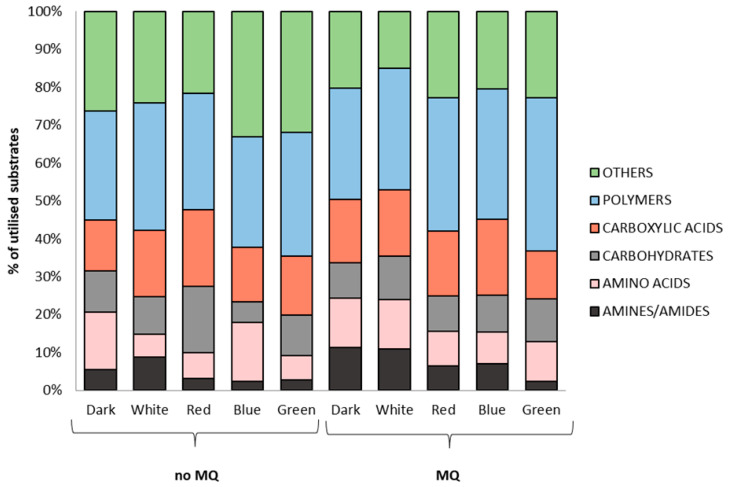
Metabolic preferences of *A. biennis* towards particular carbon and energy sources when grown in different light conditions and in the presence (MQ) or absence of menadione (no MQ).

**Figure 5:**
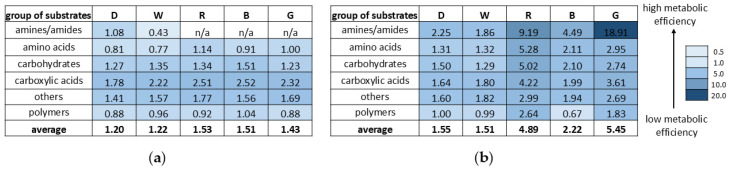
Theoretical metabolic efficiency of *A. biennis* in different light conditions and in the absence (**a**) and presence (**b**) of menadione defined as the ratio between the normalised values of mitochondrial activity (490 nm) and the mycelial growth rate (750 nm); n/a—not applicable.

Next, the comprehensive Biolog-based analysis allowed for a demonstration of the effect of light (white, red, blue, and green light, darkness) in combination with/without menadione on the metabolic capabilities of *A. biennis*. Using FF Panels, it was possible to determine the metabolic profile of the basidiomycete studied, which varied greatly during fungus growth and appeared to be dependent on the culture conditions used. In particular, the influence of menadione seemed to be evident (Figure 2). Regardless of the growing conditions, *A. biennis* could not utilise sebacic acid and monomethyl succinate. The fungus demonstrated outstanding capabilities to catabolise arbutin, salicin, and α-ketoglutaric acid, as shown in the heat map (Figure 2). Interestingly, *p*-hydroxyphenylacetic acid appeared to be a highly utilised carbon source but only in the non-induced cultures. In the presence of menadione, the compound was utilised only in the red light variant. The absence of menadione in the culture medium resulted in the preferential utilisation of mono-, di-, and trisaccharides, i.e., gentobiose, maltose, trehalose, cellobiose, glucose, and maltotriose, by *A. biennis*. This phenomenon, in addition to the cultivation under red light, is not generally visible in menadione-induced stress conditions. In turn, galactose and fucose are metabolised only in the presence of menadione. The varied metabolic capabilities of *A. biennis* were reflected by the index of overall metabolic activity (AWCD), which was 49% lower when menadione was not used. The application of menadione increased the AWCD, especially in the green and red light conditions. Significant differences were demonstrated in the richness values. The fungal culture supplemented with menadione and growing in the red light showed the highest metabolic activity, which was indicated by its capacity to utilise 35 out of the 95 carbon sources, as measured at 490 nm. In the absence of menadione in the culture medium, the observed quantitative variations were considerably less marked. The measured ability to grow on individual carbon sources (OD of 750 nm) proved to be much less variable and dependent on the growing conditions (Figure 3). In turn, the metabolic preferences of *A. biennis* towards the utilisation of a specific group of carbon sources were shown to be correlated with the presence of menadione and light conditions (Figure 4). *A. biennis* exhibited preferences for the utilisation of polymers (29–40%). In the absence of menadione, the miscellaneous C-sources were also frequently metabolised when blue and green light was applied. In general, amines/amides appeared to be the least frequently utilised group of substrates, except for the menadione oxidative stress-induced dark and white variants, where 11% of amines/amides were metabolised. It should also be noted that *A. biennis* had a low ability to grow on carbohydrates, which accounted for 5% of all the substrates, under blue light. The analysis of the 490/750 ratios between the normalised values of mitochondrial activity (OD of 490 nm) and biomass production (OD of 750 nm) by *A. biennis* in the induced and non-induced cultures in the different light conditions also showed interesting and diverse metabolic efficiency of this fungus (Figure 5). The theoretical metabolic efficiency in the non-induced cultures was quite similar, ranging from 1.20 to 1.53, while the use of menadione apparently lowered the metabolic efficiency in the green and red light conditions (5.45 and 4.89 on average, respectively), and polymers were the most efficiently metabolised substrates.

**Figure 6:**
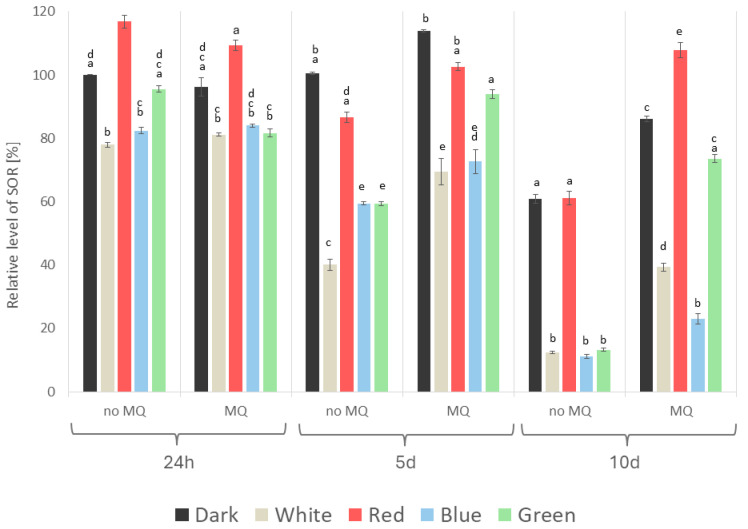
Relative level of the superoxide anion radical (SOR) detected in *A. biennis* mycelium in different lighting conditions, in menadione-induced (MQ) and non-induced (no MQ) cultures at 24 h, and on days 5 and 10 after the chemical stress stimulation; bars represent means with error bars denoting standard deviation (SD) from three measurements (n = 3); and the letters above the result bars indicate statistically homogeneous groups for each treatment, according to Tukey’s HSD test.

In the next stage of the experiment, we aimed to evaluate the effect of different lighting conditions with simultaneous applications of the oxidative stressor (menadione) on selected biochemical markers of the white rot fungus *A. biennis* grown in stationary cultures on a liquid medium. The level of superoxide anion radical (SOR) determined relative to the control, which was a menadione non-induced culture of *A. biennis* mycelium cultivated in the dark (24 h after the menadione treatment), appeared to be dependent on both the light variant used and the presence of the chemical stressor (Figure 6). In general, the highest relative levels of SOR were recorded in cultures grown in darkness and red light throughout the proposed experimental period. The highest value in the darkness variant (113.92%) was reached on day 5 after the menadione application. In contrast, in the case of red light, the relative level of SOR reached 116.78% on the first day in the menadione non-induced cultures, which was the maximum value throughout the entire measurement period. Notably, on average, the highest SOR levels were recorded in red light; they were as high as 106% in the menadione-supplemented cultures and 98% in the darkness variant. The effect of menadione was particularly evident in the later incubation period. Five and ten days after the chemical stress stimulation of *A. biennis*, the relative level of SOR was much lower in the menadione-treated cultures, ranging from 100.53 (darkness) to 40.12 (white light) on day 5 and from 61.11 (red light) to only 11.04% (blue light) on day 10 of the experimental period, compared to the control. At the same time, it should be mentioned that there was also a clear decrease in the level of SOR on day 10 in the menadione-induced cultures, which was particularly evident when white light and blue light were used. What is more, in these experimental variants, the lowest SOR level was noted compared to the other light conditions, irrespective of the incubation time and the presence or absence of the chemical stressor. Moreover, the lowest average level of SOR (only 43%) was recorded in the menadione-unstimulated cultures under white light.

**Figure 7:**
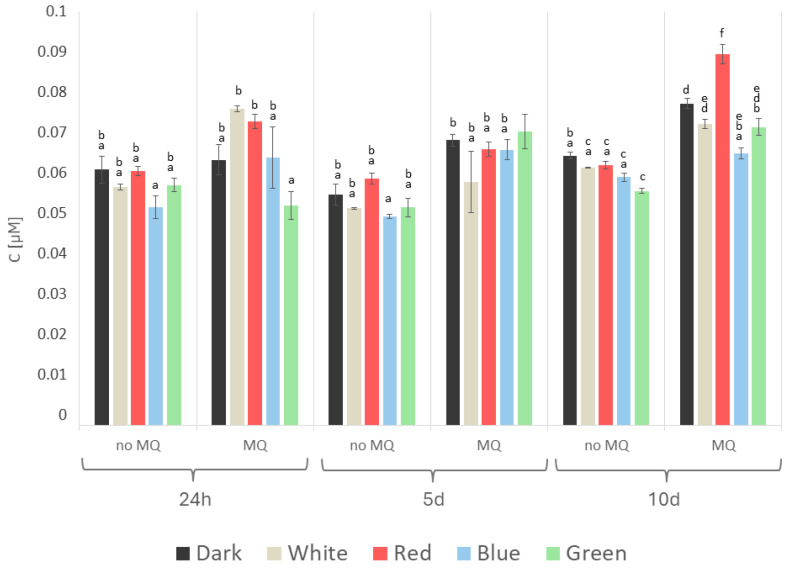
Content of phenolic substances (PHC) detected in *A. biennis* mycelium in different lighting conditions, in menadione-induced (MQ) and non-induced (no MQ) cultures at 24 h, and on days 5 and 10 after the chemical stress stimulation; bars represent means with error bars denoting standard deviation (SD) from three measurements (n = 3); and the letters above the result bars indicate statistically homogeneous groups for each treatment, according to Tukey’s HSD test.

The synergistic effects of the light and menadione treatment were much less significant in the measurements of phenolic compounds (Figure 7). The presence of menadione slightly increased the phenolic concentration in the *A. biennis* cultures as early as 24 h after the menadione supplementation, reaching its highest level on day 10 after the stress stimulation, with an average of 0.075 µM, regardless of the light conditions applied. The highest concentration of phenolics was measured in the red light variant, and this parameter was 0.089 µM, while the lowest phenolic content was shown on day 5 by the non-induced samples cultivated under blue light (0.050 µM).

The determination of the antioxidative potential of *A. biennis* cultured in different light conditions and after the oxidative stress induction showed a variable and uncorrelated effect of these potential stressors on the ability of the fungal preparations to scavenge the DPPH^•^ radical (Table 1). However, the effect of the culture time and lighting conditions on the antioxidative effect was visible and statistically significant. The strongest stimulation was observed on the first or fifth day after the chemical stress stimulation. The most substantial antioxidative effect was observed on the 5th day in cultures incubated in green light, regardless of the presence or absence of menadione, and the DPPH^•^ radical scavenging capacity was as high as 39.55 and 32.47%, respectively. In turn, the lowest antioxidant potential was observed on the 10th day in the menadione-induced cultures in blue light (0.0%) and darkness (1.4%). Despite these differences, the average ability to scavenge DPPH^•^ radical in the presence and absence of menadione in the culture medium was quite similar, reaching 19.24 and 18.57%, respectively.

**Figure 8:**
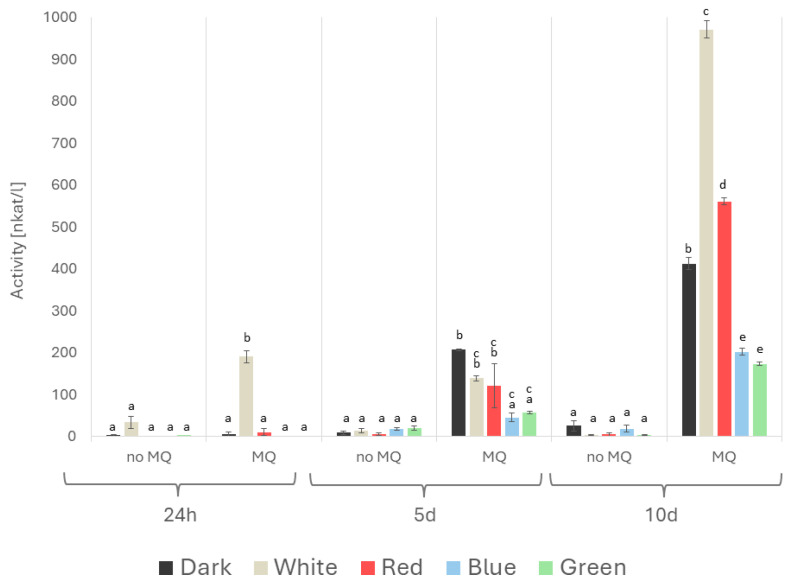
Activity of *A. biennis* intracellular (mycelial) laccase in different lighting conditions, in menadione-induced (MQ) and non-induced (no MQ) cultures at 24 h, and on days 5 and 10 after the chemical stress stimulation; bars represent means with error bars denoting standard deviation (SD) from three measurements (n = 3); and the letters above the result bars indicate statistically homogeneous groups for each treatment, according to Tukey’s HSD test.

**Figure 9:**
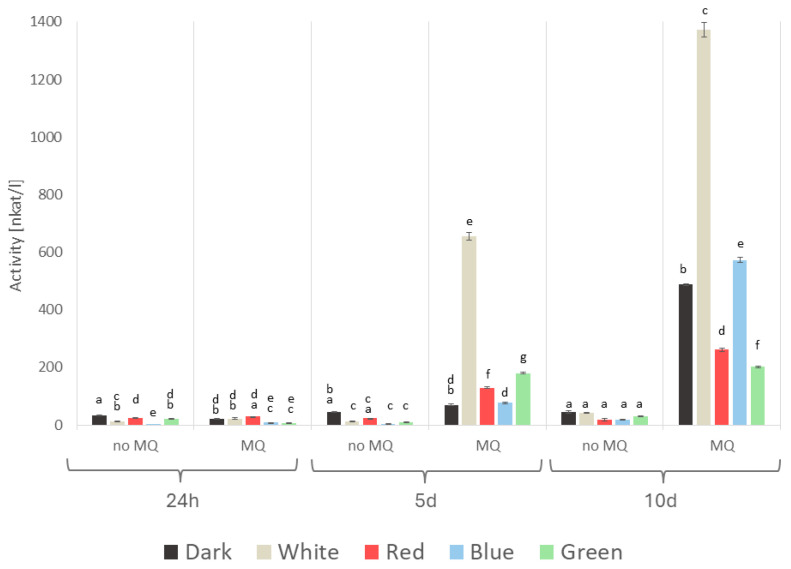
Activity of *A. biennis* extracellular laccase in different lighting conditions, in menadione-induced (MQ) and non-induced (no MQ) cultures at 24 h, and on days 5 and 10 after the chemical stress stimulation; bars represent means with error bars denoting standard deviation (SD) from three measurements (n = 3); the letters above the result bars indicate statistically homogeneous groups for each treatment, according to Tukey’s HSD test.

The effect of the abiotic stressors on the enzymatic parameters of *A. biennis* was found to vary considerably. The activities of laccase, both extra- and intracellular, measured spectrophotometrically, appeared to be strongly menadione-dependent (Figure 8 and Figure 9). In both cases, the strongest stimulation was observed on day 10 of the experimental period in the menadione-induced cultures. The highest activities were determined on that day in the white light variant; they were 971 and 1373 nkat/L for the intracellular and extracellular enzymes, respectively. Notable non-specific extracellular laccase activity reaching 654 nkat/L was also observed on the fifth day. In contrast, the lowest laccase activities of 173 nkat/L (intracellular) and 201 nkat/L (extracellular) were observed in the case of green light. Furthermore, a decline in fungal activity was evident on both day 1 and day 5 in the menadione non-exposed cultures.

**Figure 10:**
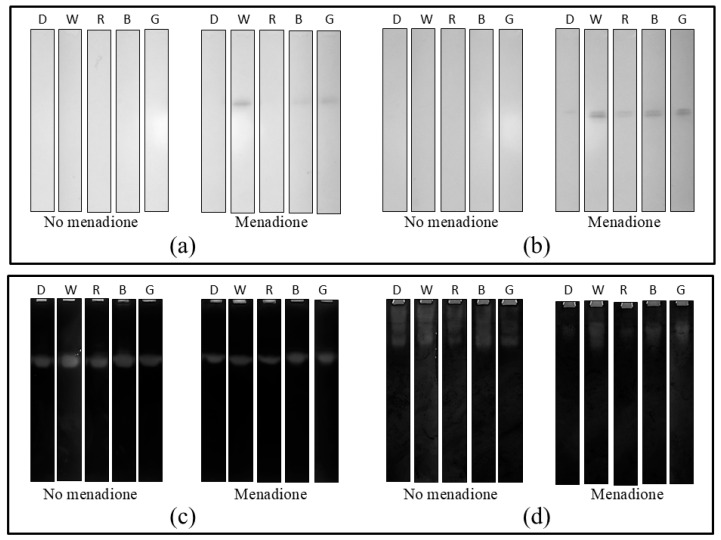
Zymographic analysis of intracellular LAC (**a**), extracellular LAC (**b**), CAT (**c**), and SOD (**d**) activity in *A. biennis* in different lighting conditions (D—darkness, W—white, R—red, B—blue, and G—green light) and in menadione-induced and non-induced cultures at 24 h (CAT and SOD) and 5 days (LAC) after the chemical stress stimulation.

The results of the zymographic analyses of laccase activity performed for *A. biennis* cultured in different light conditions in the presence and absence of menadione on day 1, 5, and 10 after the induction also confirm the above-mentioned relationships. Since the data from day 5 were considered the most significant and discriminatory, only these results are presented (Figure 10a,b and Figure S1). The zymograms obtained clearly demonstrate the presence of bands indicating laccase activity. As previously observed, the highest activities of this enzyme, i.e., the most intense activity bands, were obtained in the case of the menadione-induced cultures grown under white light. Furthermore, for extracellular laccase, an additional activity band corresponding to the slower migrating laccase isoform was observed. In contrast, the lowest intensity of the bands for intracellular laccase, or even their absence, was found in the darkness variant. However, no bands corresponding to laccase activity were found in the zymograms in the menadione non-induced *A. biennis* cultures.

A completely opposite trend was observed for catalase (CAT) and superoxide dismutase (SOD) activities obtained 24 h after the menadione-induced stress; their synthesis was generally higher in *A. biennis* cultures that were not subjected to induction with menadione (Figure 10c,d and Figure S1). However, as in the case of laccase, the intensity of the bands corresponding to the activity of both enzymes was higher in the white light conditions. It should be noted that the comparable intensity of SOD bands was also observed in the blue light and dark variants. In this case, a difference in the enzyme isoform profiles was also visible. The menadione addition caused a loss of activity of the slowest migrating isoform.

**Figure 11:**
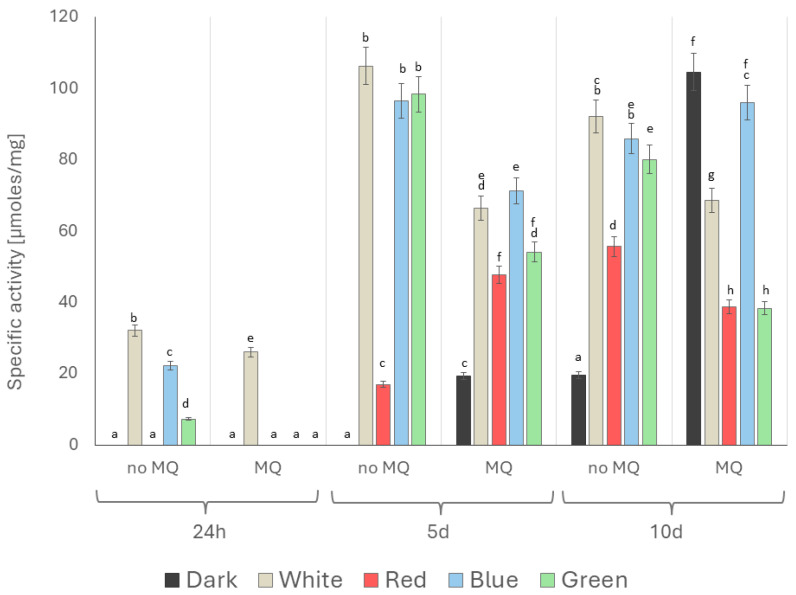
Chymotypsin-like (CHT-L) activity of *A. biennis* 26S proteasomes under different light conditions and in menadione-induced (MQ) and non-induced (no MQ) cultures measured 24 h and on days 5 and 10 after the menadione stimulation; bars represent means with error bars denoting standard deviation (SD) from three measurements (n = 3); and the letters above the result bars indicate statistically homogeneous groups for each treatment, according to Tukey’s HSD test.

The specific chymotrypsin-like (CHT-L) activity of *A. biennis* 26S proteasomes measured at 24 h and on days 5 and 10 after the induction of oxidative stress in the dark, white, red, blue, and green lighting conditions was light- and menadione-dependent. There was an apparent increase in proteasomal activity that progressed with the time of fungal cultivation, and clear stimulation of the CHT-L activity of proteasomes was visible on day 10 after the induction, irrespective of the culture conditions. In turn, the lowest level of activity, ranging from 0 in the dark, red, blue, and green light variants to 26.06 µM/mg in the white light conditions, was determined 24 h after the menadione induction. In contrast, the highest proteasomal activities were recorded on day 5 in white, green, and blue light in the menadione non-induced *A. biennis* cultures (106.29, 98.30, and 96.49 µM/mg, respectively). Another interesting effect was observed on day 5 after the oxidative stimulation. The introduction of menadione to the culture media caused the appearance of proteasomal activity in samples grown in the dark. It should be emphasised that the CHT-L activities measured in the dark were the lowest in this measurement variant, whereas they appeared to be the highest and reached 104.49 µM/mg in all the menadione-induced culture variants on day 10. Proteasomal activity in the cultures exposed to white light was also clearly evident, irrespective of the day and the presence or absence of the redox cycler (Figure 11).

## 3. Discussion

Given the ability to produce various bioactive compounds, white rot fungi are organisms with a wide range of practical applications [22]. However, the ability to withstand abiotic stress is crucial, as it both allows successful competition in nature and reveals their biotechnological potential. Fungi have developed sophisticated defence mechanisms against oxidative stress, utilising both enzymatic and non-enzymatic scavengers. Detoxification-related macromolecules are essential because lignocellulose degradation processes produce free radical compounds, peroxides, low-molecular-weight aromatics, terpenoids, resins, and tannins that can be toxic to organisms [18]. One of the representatives of white rot fungi that has been previously studied in relation to stress induced by paraquat and heavy metals is *A. biennis* [18,23]. In the transcriptome of *A. biennis*, the presence of transcripts encoding photoreceptors and associated enzymatic proteins, known to be involved in the stress response, has been demonstrated in this work.

The present study investigated global metabolic changes in *A. biennis* triggered by menadione-induced stress (chemical stressor) and different lighting conditions, i.e., red, blue, green, and white light and darkness (physical stressor). The Biolog System’s phenotyping approach facilitated the investigation of the metabolic capability of *A. biennis* in the presence of the aforementioned stress factors. A similar concept was used in environmental studies dealing with the metabolic response of microbial communities to such stress factors as heavy metals, hydrocarbon contamination, high salinity, or high soil pH [24,25,26]. It has also been successfully used to study the effects of metals and pH on the marine saprobic fungus *Aspergillus terreus* [27] and light on *Cerrena unicolor* [8]. Arbutin, salicin, and α-ketoglutaric acid were the universal carbon sources for *A. biennis*. While the first two may provide energy for the growing fungus, α-ketoglutaric acid has been identified as an antioxidant that plays a vital role in metabolism and nitrogen balance [28]. Its metabolisation may be indicative of general cellular stress. In particular, the fungal metabolism seemed to be strongly menadione-dependent. *p*-Hydroxyphenylacetic acid, found in pathways related to lignin biodegradation, appeared to be a highly utilised carbon source by *A. biennis* but only in the non-induced variants. The universality of the use of this compound as a carbon source in different lighting conditions has previously been demonstrated in *C. unicolor*. However, *C. unicolor* growing in the dark showed the highest metabolic activity [8]. In the present study, no such light dependency was observed, and the substrate richness values for *A. biennis* measured at 490 nm in the non-induced conditions demonstrated no significant differences. In turn, in the menadione-induced cultures, the aforementioned *p*-hydroxyphenylacetic acid was highly utilised only in the red light variant. The *A. biennis* mitochondrial activity measured at 490 nm, as the ability to reduce NBT, differed significantly from the mycelial growth rate (OD of 750 nm) in the presence of menadione, which indicates more intense cellular metabolism and mitochondrial activity and is not reflected in the mycelium growth capacity. The theoretical metabolic efficiency, which is a response to stress in the fungal growth environment, also appeared to confirm these results. This parameter also reflects the saprotrophic lifestyle of the representative of wood-degrading fungi and shows its preference towards polymer degradation. It is suggested that the stress response in fungi strongly depends on the compounds present in the fungal growth environment [29,30].

The antioxidant status of cells in living organisms is a balance between the production of reactive oxygen species and the level of both enzymatic and non-enzymatic antioxidant substances. Consequently, it is important to evaluate the antioxidant status of the cells under investigation. The recognised elements of these mechanisms include phenolic substances and the overall antioxidant potential [12,31]. In order to conduct a comprehensive biochemical analysis of the changes induced by the interaction of different oxidative stress factors, enzymatic (CAT, SOD, LAC, and proteasomal activity) and non-enzymatic parameters (superoxide anion radical, phenolic substances, and antioxidant potential) were measured in the *A. biennis* cultures. In general, the level of phenolic compounds (PHCs) in the treatment variants did not show very significant differences. Since many phenolic compounds have antioxidant properties and can neutralise heavy metals, the ability to synthesise these compounds may alleviate the effects of stress, helping to scavenge free radicals [32,33,34]. At the same time, the ability of fungi to produce phenolic compounds demonstrates their biomedical potential [35]. For some white-rot fungal organisms, such as *Lentinus crinitus*, phenolic compounds exhibit wavelength-dependent antioxidant activity, with light quality influencing both their production and their efficiency in scavenging ROS. Cultivation under red light resulted in the highest levels of phenolic compounds (e.g., benzoic and gallic acids) and antioxidant capacity compared to exposure to blue/green light. It was suggested that red light may act as a mild stressor, triggering the biosynthesis of phenolic compounds as part of the fungus’s antioxidant defences, while blue/green light prioritised primary metabolism and fungus development [36]. In this study, the presence of menadione increased the phenolic concentrations. The highest level of PHC was measured in the menadione-induced cultures of *A. biennis* grown in the red light variant, which may result from the fungal cell dealing with elevated stress conditions, compared to other variants. When analysing the ability of *A. biennis* to metabolise particular substrates, its above-average ability to oxidise *p*-hydroxyphenylacetic acid in the menadione non-induced conditions was evident. Meanwhile, in the presence of menadione, these properties were much weaker, and only red light contributed to the increased fungal ability to utilise this compound. The production of SOR, which is particularly important for wood-degrading fungi for redox cycles in lignin degradation and nutrient acquisition and can also contribute to oxidative stress [37], was maintained at different levels depending on the *A. biennis* cultivation variant, which may result from remodelling of the fungal intracellular biochemical defence system [4]. Fungal photoreceptors exhibit distinct roles in SOR generation, with specificity tied to their light sensitivity and downstream signalling pathways. In *Aspergillus nidulans*, phytochrome (red light photoreceptor) mutants show increased resistance to oxidative stress, suggesting phytochromes modulate ROS balance by influencing antioxidant pathways, thus indirectly affecting SOR generation by regulating secondary metabolism and stress response genes. The involvement of the FphA phytochrome of *A. nidulans* in blue-light sensing and ROS generation in response to intense blue light was also proven [38,39]. Opsins (green light photoreceptors) such as CarO in *Fusarium fujikuroi* may influence SOR levels by modulating cellular redox states or proton-pumping activity. However, the signalling pathway remains unexplored [40,41]. Exposure to red and white light has also been shown to affect the stress response of *Monilinia fructicola* by influencing the metabolism of reactive oxygen species and increasing H_2_O_2_ production [42]. Although the highest SOR level was found in the menadione non-induced red light *A. biennis* cultures and the darkness variant supplemented with menadione, it is apparent that, in general, the highest levels of SOR were detected in cultures grown in the red light and dark conditions. The increased sensitivity of the fungus to red light, manifested by the elevation of some biochemical markers of stress, can be explained by a link between red light and stress signalling identified in *A. nidulans*. In this fungus, the high osmolarity glycerol (HOG) MAP kinase pathway is required to transmit a red light signal to the nucleus. At the same time, the HOG pathway is the central module of stress signalling responsible for reactive oxygen species (ROS) detection [43]. Similarly, the phytochrome PHY-2 in *N. crassa* interacts directly with the HOG pathway via a phosphorelay system involving YpdA and SskA, which activate the MAP kinase Hog1, regulating stress adaptation and response [44]. However, the *N. crassa* retinal-binding opsin NOP-1, which regulates developmental genes and lacks a direct link to the HOG pathway, can modulate the activity of the White Collar Complex (WWC; the central blue-light photoreceptor in fungi), presumably through the light-dependent activation of a putative repressor of the WCC [44,45]. Hog1 activation has also been demonstrated to be a critical component of the ROS-generated stress response in *C. albicans* [46]. A link between light and stress signalling was also discovered in *Botrytis cinerea* and *C. unicolor* [30,43]. Menadione supplementation usually increases the relative level of SOR in fungi [14]. In the present study, we observed the effect of the overlapping of two stress factors, which probably caused changes in this correlation.

Lignolytic extracellular enzymes of white rot fungi, i.e., LAC, are known to catalyse free radical formation [12,47]. It has also been postulated that enzymes lacking direct lignin-degrading ability, such as CAT or SOD, are essential in lignolysis as protective agents and a source of such substrates as hydrogen peroxide [19]. Therefore, changes in the levels of these enzymes in stress conditions can affect protein turnover in the cell and be reflected in the level of protease synthesis [30]. The detection of proteolytic activity associated with *A. biennis* 26S proteasomes revealed a marked progression in the rate of substrate digestion at the late stage of fungal growth. The role of ATP-dependent proteolysis in cultures exposed to white light was also clearly evident, irrespective of the day and the presence or absence of menadione. The stimulation of the CHT-L activity by white light has also been observed in *C. unicolor* [30,48]. The laccase activity seemed to be strongly induced by menadione, and the lighting conditions used did not play a significant role in enhancing its level. A similar lack of light dependence was observed previously for *C. unicolor* laccase [49]. The effect of light was only seen upon the addition of menadione, which appeared to significantly increase laccase activity under white light. Under these conditions, intracellular and extracellular laccase activities increased by 5.6- and 6.8-fold, respectively, compared to the corresponding activities under menadione-induced green light, which showed the lowest levels on the tenth day. The observed increase in enzyme activity stimulated by menadione and white light—both of which are inexpensive and non-toxic—suggests their potential use in industrial methods for *A. biennis* laccase production, which often rely on costly chemical inducers such as aromatic and phenolic compounds [50,51]. The ability to stimulate laccase activity through synergistic action is important given the potential biotechnological applications of this enzyme, particularly in medical and industrial biotechnology [3,4]. The catalase and superoxide dismutase activities were found to be induced by the culture conditions used, but their synthesis appeared to be higher in the menadione non-induced cultures of *A. biennis*. This is of particular interest given that the effect of both light and menadione on enzyme synthesis depends on the fungal species tested. As demonstrated in the studies of [49,52], the incorporation of menadione and red light has been shown to result in a substantial increase in SOD activity in yeast-related *Eremothecium gossypii* and *C. unicolor*, respectively. It is evident that the metabolic capacity of fungi can be modulated and manipulated to align with specific requirements by subjecting the cell to stress. This approach has been proposed to stimulate the biotechnological potential of wood-degrading fungi [3,4,16,53]. Cultivating mycelium under specific light conditions also appeared to be a promising strategy for controlling its chemical composition and biomass yield [36].

We conclude that the response of *A. biennis* to light- and menadione-induced oxidative stress is complex and multidirectional, with the effect depending on the parameter studied. Nevertheless, a noticeable synergistic effect of red light and menadione on the overall metabolic activity as well as the ability to catabolise individual carbon sources and the concentration of phenolic compounds can be observed. The presence of menadione also positively influenced the stimulation of laccase activity. Considering the cross-talk between oxidative stress and light response pathways in fungi, the observed biochemical changes are a resultant phenomenon of the action of these two factors. These data may provide a basis for the efficient, eco-friendly, and simple improvements of the industrial and medicinal applications of *A. biennis*.

## 4. Materials and Methods

### 4.1. Fungal Strain and Cultivation

The *Abortiporus biennis* FCL123 (KC862285) strain from the Fungal Culture Collection (FCL) of the Department of Biochemistry, Maria Curie-Sklodowska University in Lublin, Poland [54], was used in this work. Mycelium-overgrown agar fragments (5 mm^2^) were used to inoculate 100 mL of Lindeberg–Holm (LH) liquid medium [55]. Next, the inoculated culture flasks were incubated stationarily for 10 days at 28 °C and then used for further experiments.

### 4.2. Analysis of the Expression Profile of Genes Encoding Putative Oxidative Stress Response Proteins

In search of the expression profile of genes putatively involved in the oxidative stress response, we manually reanalysed the gene expression data during *A. biennis* cultivation obtained in the RNA-seq experiment, deposited in the Sequence Read Archive database of NCBI under BioProject PRJNA450312, and described elsewhere [21].

### 4.3. Evaluation of the Effect of Lighting Conditions and Menadione-Induced Oxidative Stress on the Growth and Metabolic Profiles of A. biennis Based on Phenotypic Analysis Using Biolog FF Panels

The impact of different lighting conditions on *A. biennis* metabolic abilities to utilise carbon substrates was measured using FF MicroPlates, Biolog, Hayward, CA, USA (95 carbon sources). Cell suspensions were prepared from *A. biennis* FCL123 mycelia cultured in LH medium [55], homogenised in a disperser homogeniser T18 basic ULTRA-TURRAX (IKA, Staufen, Germany), and transferred into sterile inoculating fluid (FF-IF, Biolog, Hayward, CA, USA). The turbidity of the suspension was unified and adjusted to 75% of transmittance, according to the manufacturer’s protocol. The FF plates were inoculated with the cell suspension, and the mycelia were then cultivated at 28 °C in incubators equipped with LED illumination cassettes (KT 115, Binder, Tuttlingen, Germany) as described previously [8]. Continuous lighting conditions (20 lux) were provided throughout the entire period of *A. biennis* cultivation. The following lighting variants and conditions were applied: white (W; 4000–4750 K, 2.88 µmol·m^−2^·s^−1^), green (G; 510–520 nm, 2.85 µmol·m^−2^·s^−1^), blue (B; 465–470 nm, 2.62 µmol·m^−2^·s^−1^), red (R; 620–625 nm, 3.52 µmol·m^−2^·s^−1^) light and dark (D; 0.0 µmol·m^−2^·s^−1^). On the 5th day of the fungus incubation, menadione (MQ; 2-methyl-1,4-naphtoquinone) was added to a final concentration of 1.0 mM to plates subjected to evaluation of menadione-induced oxidative stress. The changes in the wells were measured at 490 nm (mitochondrial activity) and 750 nm (mycelial growth) every 24 h (Infinite 200 Pro microplate reader, Tecan, Crailsheim, Germany), which provided both amplification and quantitation of the phenotype. All measurements were performed in triplicate in two biological replications. The most consistent readings came from 8-day-old Biolog MPs, and these were used in the analyses. The average values of colour development (AWCD) from all readings for each compound recorded at 490 nm and the average values of density development (AWDD) measured at 750 nm were used for data analysis [56]. Analysis of variance (ANOVA) was used to determine the differences in the rate of utilisation of a particular substrate in relation to the light or light and menadione variant used. Post hoc analyses were performed using Tukey’s test (HSD). All data were presented as 95% confidence intervals. Statistical significance was established at *p* < 0.05. Statistical analyses were performed using the STATISTICA 13.0 (StatSoft, Inc., Tulsa, OK, USA) software package.

### 4.4. Preparation of Experimental Cultures, Induction of Stress Conditions, and Biochemical Analysis of A. biennis

*A. biennis* mycelia cultured as described above were homogenised in a disperser homogeniser (IKA, Staufen, Germany) and used (2.5% (*v*/*v*)) for the inoculation of 25 mL Erlenmeyer flasks containing 10 mL of LH medium. The *A. biennis* cultures were then grown at 28 °C in incubators (KT 115, Binder, Germany) equipped with illumination LED cassettes for 10 days. The following lighting conditions were applied: white (W; 4000–4750 K, 2.88 µmol·m^−2^·s^−1^), green (G; 510–520 nm, 2.85 µmol·m^−2^·s^−1^), blue (B; 465–470 nm, 2.62 µmol·m^−2^·s^−1^), red (R; 620–625 nm, 3.52 µmol·m^−2^·s^−1^) light and darkness (D; 0.0 µmol·m^−2^·s^−1^). Continuous lighting conditions (20 lux) were provided throughout the entire period of *A. biennis* cultivation. On the 10th day of incubation, oxidative stress was induced by adding menadione to a final concentration of 1.0 mM [14]. The mycelium (intracellular preparation) and post-culture fluid (extracellular preparation) were then collected at 24 h and on the 5th and 10th days after the menadione and light treatment. A control experiment (no menadione in the culture medium) for each lightning condition was also performed. All experiments were performed in triplicate.

#### 4.4.1. Preparation of Crude Extracts

Intracellular extracts were prepared using mycelium thoroughly rinsed with cold distilled water and homogenised on ice using a Potter-S glass homogeniser AG3707, Sartorius AG, Gottingen, Germany (2 min processing) in 5 mL of ice-chilled distilled water. Immediately after the homogenisation process, each sample was centrifuged (10,000× *g*, 15 min, 4 °C). Extracellular extracts (post-culture fluids) were obtained from cultures cultivated in different lighting variants and in menadione-induced or non-induced conditions at 24 h and on the 5th and 10th day after the stress stimulation. Each sample was filtered through a Miracloth membrane. All extracts were then aliquoted, frozen, and stored at −70 °C until further enzymatic (laccase, catalase, superoxide dismutase, and 26S proteasomal activity) and non-enzymatic (concentration of proteins, phenolic compounds, and the relative level of superoxide anion radical) analyses.

#### 4.4.2. Detection of the Relative Level of Superoxide Anion Radical (SOR)

The measurement of superoxide anion radical levels was based on the reduction of yellow nitrotetrazolium blue (NBT) to the blue diformazane complex in alkaline conditions by free radicals present in the tested samples [57]. The colour intensity is proportional to the content of the superoxide anion radical in the sample. The detailed procedure was described previously by Jaszek et al. [20]. The SOR level was expressed as a percentage (%) relative to the control, i.e., 24-h fungal culture incubated in the dark without the addition of menadione, which was set as 100%.

#### 4.4.3. Determination of Phenolic Substances (PHCs)

The concentration of phenolic substances was measured spectrophotometrically based on the ability of phenolic compounds to couple with sulphanilamide, resulting in the formation of a diazo derivative (DASA test) [58]. The detailed procedure was described previously by Jaszek et al. [20]. The concentration of phenolic compounds (PHCs) was expressed as μmol of vanillic acid equivalent per ml for the post-culture liquids and per mg of protein for the mycelia, based on a calibration curve prepared for known concentrations of vanillic acid.

#### 4.4.4. Determination of Antioxidative Properties

The antioxidant properties were measured as free radical (DPPH^•^; 1,1-diphenyl-2-picrylhydrazyl) scavenging activity as described in the DPPH^•^ assay [59]. The detailed procedure was described previously by Jaszek et al. [20]. The capability to scavenge the DPPH^•^ radical was calculated according to the following Formula (1):

(1) $${DPPH}^{•}\;antioxidative\;effect\left(\%\right)=(A\mathrm{control}-A\mathrm{sample})/A\mathrm{sample}\times 100$$

where Acontrol is the absorbance of the control solution, and Asample is the absorbance of the tested sample.

#### 4.4.5. Laccase (LAC) Assay

Using syringaldazine as a substrate [60], laccase activity was detected spectrophotometrically at 525 nm. Enzyme and substrate blanks were included. One unit of laccase activity was defined as the amount of the enzyme needed to catalyse the production of one nanomole of coloured product (quinone, ε = 65,000 M^−1^cm^−1^) per second at 25 °C and pH 5.2. The activity was expressed as nkat/L.

#### 4.4.6. Zymographic Analysis of Catalase (CAT), Superoxide Dismutase (SOD), and Laccase (LAC) Activity

Native-PAGE electrophoretic analysis of enzyme activity was performed using 15 μg of protein per lane on a 10% separating gel and a 4% stacking gel according to Laemmli [61]. Electrophoresis was performed in non-denaturing conditions at 4 °C and 145 V. After electrophoretic separation, the SOD activity was detected based on the Beyer and Fridovich (1987) analytical method [62]. The ferricyanide negative staining method was used to determine catalase activity [63]. For electrophoretic detection of laccase activity, immediately after sample separation, the gels were stained with a 0.01% guaiacol (2-methoxyphenol) solution in 0.1 M citrate-phosphate buffer, pH 5.2, for 30 min, until bands corresponding to enzyme activity appeared [64]. The imaging and analysis of electropherograms were performed using the G: Box gel documentation system (Syngene, Frederick, MD, USA). All zymographic analyses were performed in triplicate.

#### 4.4.7. Measurement of Proteasome Peptidase Activity

The intracellular extracts were concentrated using ultrafiltration units (Vivaspin 20, 300,000 MWCO, PES, Sartorius AG, Gottingen, Germany) at 4 °C. Next, the high-molecular-weight fractions obtained were tested for 26S proteasome chymotrypsin-like (CHT-L) activity as described previously in [30]. Briefly, 100 μM of the fluorogenic peptide substrate Suc-LLVY-AMC (Suc-Leu-Leu-Val-Tyr-7-amido-4-methylcoumarin in DMSO), 100 mM Tris-HCl buffer (pH 8.0), 2 mM ATP, and 5 mM MgCl_2_ in a total volume of 100 μL were incubated at 37 °C for 2 h with shaking. The modified stopping procedure based on 10% SDS (*w*/*v*) and 100 mM Tris-HCl buffer, pH 9.0 [65,66], was applied as described previously in the study of [67]. A spectrofluorometer (FluoroMax-2, Horiba Scientific/Spex Industries, Edison, NJ, USA) with an excitation λ = 360 nm and an emission of λ = 440 nm was used to quantify the fluorescence of 7-amido-4-methylcoumarin (AMC) formed in the reaction. The amount of released AMC was calculated using a standard curve. An appropriate control experiment was performed to subtract the fluorescence of the culture medium for each culture condition [68]. Specific peptidase activity was expressed in micromoles of AMC released per milligram of protein (µmoles/mg) per 2 h.

#### 4.4.8. Protein Determination

The protein concentration was determined according to the Bradford method [69] with crystalline bovine serum albumin (BSA) as a standard in a range of 8–80 μg/mL, as recommended in the microassay procedure for microtiter plates. All assays were carried out in triplicate.

#### 4.4.9. Statistical Analysis

All measurements were performed in triplicate in three independent biological replications. All results are expressed as the mean ± SD (standard deviation) from three measurements (n = 3). A two-factor analysis of variance (ANOVA) was performed at each time point to investigate the effects of light and the presence of menadione on the concentration of phenolic compounds, the relative level of the superoxide anion radical (SOR), laccase activity, and chymotrypsin-like activity (CHT-L) in *A. biennis*. Post hoc analyses were performed using Tukey’s test (HSD). Statistical significance was established at *p* < 0.05. Analysis of variance (ANOVA) was used to determine the effect of lighting conditions, time, and the presence of menadione on antioxidant properties of *A. biennis*. A Student’s *t*-test with Bonferroni correction was performed, and statistically significant differences were identified at *p* = 0.05/15 ≈ 0.003. Statistical analyses were performed using the STATISTICA (data analysis software system) version 13.0 (TIBCO Software Inc., San Ramon, CA, USA).

## Figures and Tables

**Table 1:** Antioxidative effect of *A. biennis* mycelium in different lighting conditions and in menadione-induced (MQ) and non-induced (no MQ) cultures measured as the ability to scavenge DPPH^•^ radical [%] at 24 h and on days 5 and 10 after the chemical stress stimulation: results represent means with standard deviation (SD) from three measurements (n = 3); a Student’s *t*-test with Bonferroni correction is used to present the differences between the means of the variable (antioxidant ability) for different combinations of time and lighting conditions, depending on the presence of menadione; lowercase letters indicate statistically homogenous groups for different combinations of time and light, excluding the menadione according to an HSD Tukey test.

Lighting Conditions	Time Since Induction with Menadione	No MQ	MQ	No MQ vs. MQ Statistical Difference *p*-Value and Statistical Significance	No MQ and MQ Combined Groups
	24 h	29.48 ± 4.99	29.29 ± 3.61	0.9609	29.39 ± 3.90 ^c,d,e^
Dark	5 days	11.28 ± 1.92	20.89 ± 3.81	0.0175	16.09 ± 5.91 ^a,b,c,d^
	10 days	13.26 ± 2.18	1.40 ± 1.86	0.0020 *	7.33 ± 3.94 ^a^
	24 h	21.44 ± 6.08	26.32 ± 6.44	0.3941	23.88 ± 6.21 ^b,c,d,e^
White	5 days	7.00 ± 3.15	27.39 ± 3.60	0.0018 *	17.19 ± 11.57 ^a,b,c,d^
	10 days	22.14 ± 15.19	5.73 ± 1.42	0.1360	13.93 ± 13.19 ^a,b,c^
	24 h	26.90 ± 8.28	32.02 ± 0.23	0.3446	29.46 ± 5.94 ^d,e^
Red	5 days	7.92 ± 0.46	26.86 ± 6.25	0.0064	17.39 ± 11.10 ^a,b,c,d^
	10 days	14.21 ± 2.85	12.20 ± 3.20	0.4615	13.21 ± 2.93 ^a,b^
	24 h	25.85 ± 2.65	17.73 ± 2.31	0.0162	21.79 ± 4.97 ^a,b,c,d,e^
Blue	5 days	25.14 ± 5.09	22.19 ± 6.11	0.5564	23.67 ± 5.28 ^b,c,d,e^
	10 days	12.24 ± 1.84	0.00	0.0003 *	6.12 ± 6.81 ^a^
	24 h	18.38 ± 6.72	22.17 ± 5.52	0.4931	20.28 ± 5.88 ^a,b,c,d,e^
Green	5 days	32.47 ± 4.32	39.55 ± 15.85	0.4968	36.01 ± 11.09 ^e^
	10 days	10.86 ± 3.13	4.67 ± 0.39	0.0273	7.77 ± 3.94 ^a^

* statistically significant differences at *p* = 0.05/15 ≈ 0.003.

## Data Availability

The raw data supporting the conclusions of this article will be made available by the authors upon request.

## Supplementary Materials

The supporting information can be downloaded at https://www.mdpi.com/article/10.3390/ijms26125482/s1.
