# Supplementary material for: Modulation of Abortiporus biennis Response to Oxidative Stress by Light as a New Eco-Friendly Approach with a Biotechnological Perspective
Source: Int J Mol Sci. 2025 Jun 7;26(12):5482. doi: 10.3390/ijms26125482 (PMC12193379; doi:10.3390/ijms26125482)
Supplement: Supplementary file 1 [file ijms-26-05482-s001.zip › Figure S1.pdf]

Supplementary Materials

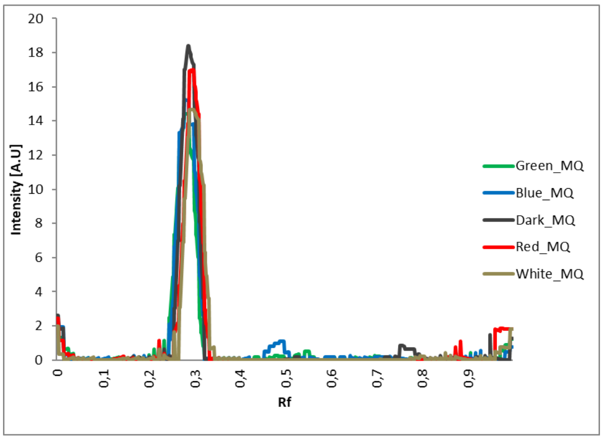

Peak height for catalase - no MQ	
Green	14.429
Blue	15.228
Dark	18.410
Red	17.004
White	14.645

(**a**)

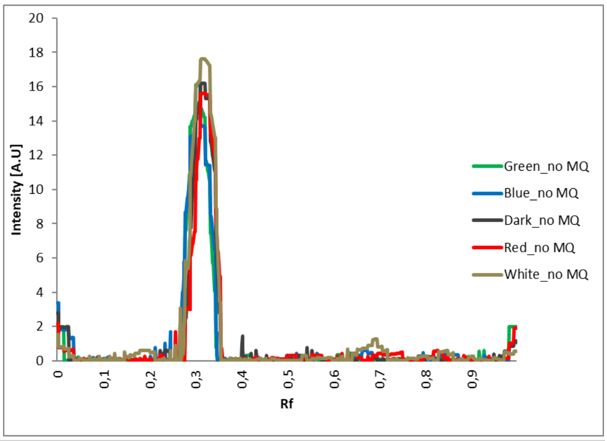

Peak height for catalase - MQ	
Green	16.113
Blue	14.521
Dark	16.190
Red	15.592
White	17.622

(**b**)

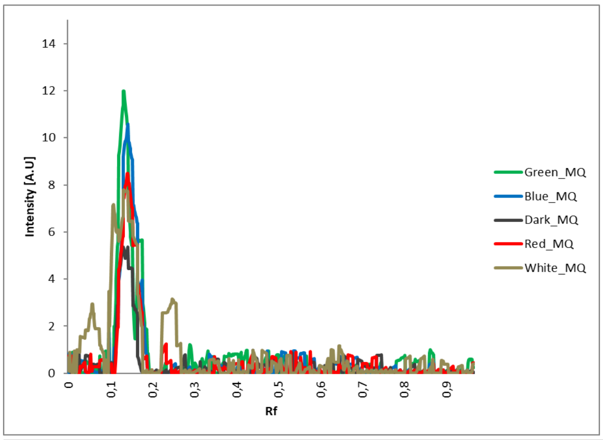

Peak height for SOD - no MQ	
Green	11.983
Blue	10.595
Dark	5.371
Red	8.491
White	2.935; 7.732; 3.066

(**c**)

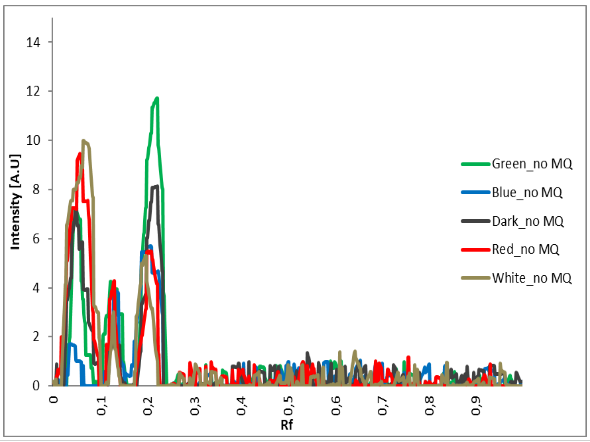

Peak height for SOD - MQ	
Green	7.197; 4.255; 11.713
Blue	1.718; 3.880; 5.686
Dark	7.234; 1.733; 8.120
Red	9.456; 4.278; 5.496
White	9.997; 3.001; 5.301

(**d**)

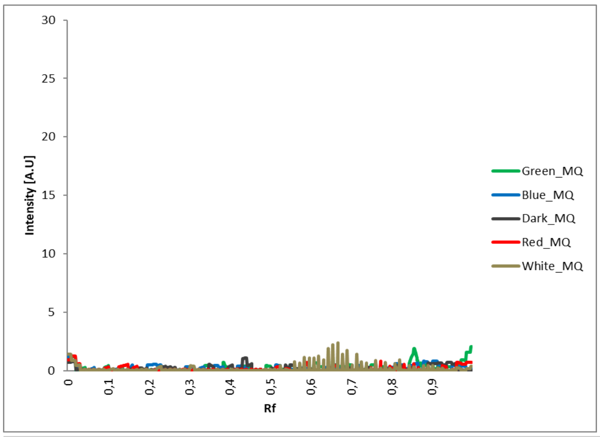

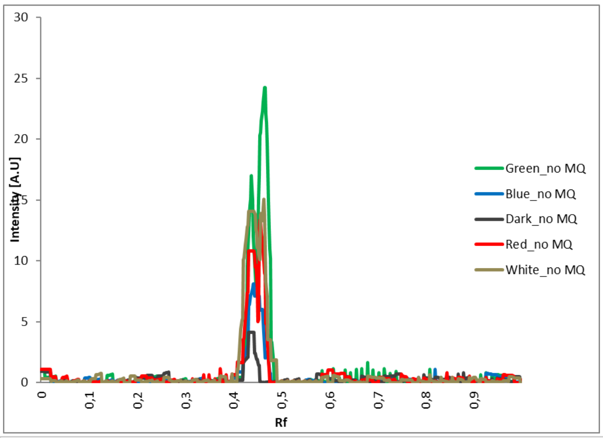

Peak height for extracellular laccase - no MQ	
Green	-
Blue	-
Dark	-
Red	-
White	-

(**e**)

Peak height for extracellular laccase - MQ	
Green	14.568; 24.234
Blue	8.125
Dark	4.136
Red	10.828; 13.259
White	14.059; 13.576

(**f**)

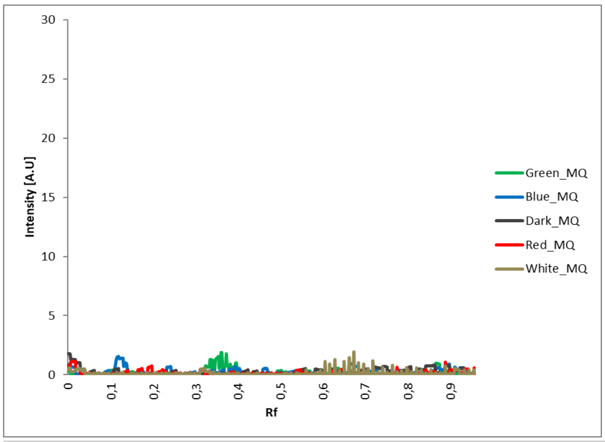

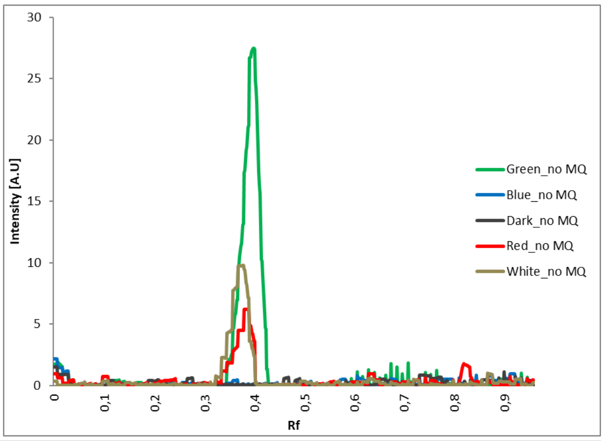

Peak height for intracellular laccase - no MQ	
Green	-
Blue	-
Dark	-
Red	-
White	-

(**g**)

Peak height for intracellular laccase - MQ	
Green	27.457
Blue	-
Dark	-
Red	6.196
White	9.765

(**h**)

Figure S1. Image analysis of zymograms showing the activity of catalase (CAT) (a, b), superoxide dismutase (SOD) (c, d), extracellular laccase (LAC) (e, f), and intracellular laccase (LAC) (g, h) in *A. biennis* under different lighting conditions (darkness, white, red, blue, and green light) and in menadione-induced (MQ – b, d, f, h) and non-induced cultures (no MQ – a, c, e, g) at 24 hours (CAT and SOD) and 5 days (LAC) following chemical stress stimulation.
